# Supplementary material for: Structures of the ATP-fueled ClpXP proteolytic machine bound to protein substrate
Source: eLife. 2020 Feb 28;9:e52774. doi: 10.7554/eLife.52774 (PMC7112951; doi:10.7554/eLife.52774)
Supplement: Supplementary file 1. [file elife-52774-supp1.doc]

**Structures of the ATP-fueled ClpXP proteolytic machine bound to protein substrate**

Xue Fei^1^, Tristan A. Bell^1^, Simon Jenni^2^, Benjamin M. Stinson^1^, Tania A. Baker^1,3^, Stephen C. Harrison^2,3^, and Robert T. Sauer^1,*^

^1^Department of Biology, Massachusetts Institute of Technology, Cambridge, MA 02139

^2^Department of Biological Chemistry and Molecular Pharmacology, Harvard Medical School, Boston, MA 02115

^3^Howard Hughes Medical Institute.

***Supplementary File 1***

| **Name** | **ClpP** | **ClpX-class1** | **ClpX-class2** | **ClpX-class3** | **ClpX-class4** | **ClpXP-class1** | **ClpXP-class2** | **ClpXP-class3** | **ClpXP-class4** |
| --- | --- | --- | --- | --- | --- | --- | --- | --- | --- |
| **PDB ID** | 6PPE | 6PP8 | 6PP7 | 6PP6 | 6PP5 | 6POS | 6POD | 6PO3 | 6PO1 |
| **EMDB ID** | 20434 | 20422 | 20421 | 20420 | 20419 | 20418 | 20412 | 20408 | 20406 |
|  | **Data collection/processing** | | | | | | | | |
| Microscope | Talos Arctica | | | | | | | | |
| Camera | K2 summit | | | | | | | | |
| Magnification | 36000X | | | | | | | | |
| Voltage (kV) | 200 | | | | | | | | |
| Total electron dose (e^-^/Å^2^) | 58 | | | | | | | | |
| Defocus range (µm) | -1.2 to -2.5 | | | | | | | | |
| Pixel size (Å) | 0.58 | | | | | | | | |
| Micrographs collected | 3657 | | | | | | | | |
| Final particles | 443717 | 151652 | 241899 | 115751 | 227257 | 151652 | 241899 | 115751 | 227257 |
| Symmetry | D7 | C1 | C1 | C1 | C1 | C1 | C1 | C1 | C1 |
| Resolution (FSC 0.143) | 3.19 | 4.12 | 4.05 | 4.28 | 3.98 | 4.12 | 4.05 | 4.28 | 4.05 |
|  | **Model composition** | | | | | | | | |
| Non-hydrogen atoms | 21840 | 16101 | 16203 | 15980 | 16006 | 26650 | 26739 | 26501 | 26527 |
| Protein residues | 2814 | 2085 | 2100 | 2068 | 2075 | 3432 | 3446 | 3412 | 3419 |
| Ligands | 0 | 6 | 6 | 6 | 6 | 6 | 6 | 6 | 6 |
|  | **Refinement** | | | | | | | | |
| Map-model CC | 0.9 | 0.77 | 0.75 | 0.78 | 0.8 | 0.76 | 0.79 | 0.75 | 0.79 |
| Map sharpening B factors (Å^2^) | -180 | -186 | -235 | -216 | -170 | -160 | -176 | -184 | -184 |
| RMSD bond lengths (Å) | 0.004 | 0.003 | 0.002 | 0.003 | 0.004 | 0.002 | 0.004 | 0.003 | 0.003 |
| RMSD bond angles (degrees) | 0.65 | 0.73 | 0.65 | 0.72 | 0.70 | 0.65 | 0.71 | 0.66 | 0.64 |
|  | **Validation** | | | | | | | | |
| MolProbity score | 0.60 | 0.94 | 0.97 | 0.91 | 0.96 | 0.90 | 0.93 | 0.84 | 0.96 |
| Clash score | 0.27 | 1.79 | 2.05 | 1.64 | 1.98 | 1.58 | 1.75 | 1.22 | 1.98 |
| C-beta deviations | 0 | 0 | 0 | 0 | 0 | 0 | 0 | 0 | 0 |
| Rotamer outliers (%) | 0 | 0 | 0 | 0 | 0 | 0 | 0 | 0 | 0 |
| Ramachandran favored (%) | 98.5 | 99.8 | 99.2 | 99.4 | 99.5 | 99.5 | 99.5 | 99.6 | 99.7 |
| Ramachandran disallowed (%) | 0 | 0 | 0 | 0 | 0 | 0 | 0 | 0 | 0 |

Table S1 Cryo-EM data collection, processing, model building and validation statistics.

|  | **subunit** | **A** | **B** | **C** | **D** | **E** | **F** |
| --- | --- | --- | --- | --- | --- | --- | --- |
| **class-1** | protein | 0.74 | 0.74 | 0.75 | 0.75 | 0.76 | 0.75 |
|  | nucleotide | 0.72 | 0.77 | 0.86 | 0.83 | 0.81 | 0.80 |
| **class-2** | protein | 0.76 | 0.78 | 0.77 | 0.77 | 0.76 | 0.74 |
|  | nucleotide | 0.78 | 0.80 | 0.84 | 0.85 | 0.84 | 0.79 |
| **class-3** | protein | 0.77 | 0.78 | 0.78 | 0.78 | 0.76 | 0.74 |
|  | nucleotide | 0.82 | 0.82 | 0.84 | 0.84 | 0.84 | 0.78 |
| **class-4** | protein | 0.80 | 0.81 | 0.81 | 0.81 | 0.81 | 0.79 |
|  | nucleotide | 0.86 | 0.87 | 0.86 | 0.84 | 0.78 | 0.73 |

Table S2 Model to map correlation coefficient (CC) of protein and nucleotides. Calculated using phenix_realspace.correlation (Adams et al., 2010).

|  | **A-B** | **B-C** | **C-D** | **D-E** | **E-F** | **F-A** |
| --- | --- | --- | --- | --- | --- | --- |
| **class-1** | 1.85 | 2.15 | 2.23 | 2.18 | 2.07 | 1.55 |
| **class-2** | 1.29 | 2.20 | 2.23 | 2.22 | 2.12 | 1.43 |
| **class-3** | 1.98 | 2.12 | 2.09 | 2.18 | 1.78 | 1.38 |
| **class-4** | 2.04 | 2.17 | 2.16 | 2.28 | 1.65 | 1.21 |

Table S3 Buried surface area (in units of 1000 Å^2^) between ClpX^∆N^ subunits. The interface in each hexamer with the smallest amount of buried surface is highlighted in gray and corresponds to the ADP-bound subunit.

| **class-1** | **R307**  **(Arg finger)** | **R370 (sensor II)** | **E185**  **(Walker B)** |
| --- | --- | --- | --- |
| A | 5.9 | 4.5 | 5.5 |
| B | 4.6 | 4.4 | 5.9 |
| C | 4.7 | 4.7 | 6.6 |
| D | 4.6 | 4.5 | 5.3 |
| E | 5.3 | 4.6 | 5.2 |
| F | 15.6 | 4.9 | 9.1 |
| **class-2** | **R307** | **R370** | **E185** |
| A | 8.4 | 4.3 | 7.9 |
| B | 4.7 | 4.1 | 5.7 |
| C | 4.9 | 4.2 | 5.2 |
| D | 4.5 | 4.3 | 6.2 |
| E | 5.9 | 4.5 | 6.9 |
| F | 15.9 | 4.2 | 7.0 |
| **class-3** | **R307** | **R370** | **E185** |
| A | 5.5 | 4.1 | 5.6 |
| B | 4.9 | 3.9 | 5.9 |
| C | 5.2 | 4.4 | 6.5 |
| D | 4.7 | 4.7 | 5.6 |
| E | 5.8 | 4.2 | 6.2 |
| F | 15.7 | 6.1 | 9.7 |
| **class-4** | **R307** | **R370** | **E185** |
| A | 5.9 | 4.0 | 5.8 |
| B | 4.5 | 4.2 | 7.9 |
| C | 4.6 | 4.7 | 6.5 |
| D | 4.6 | 4.4 | 5.4 |
| E | 8.7 | 4.6 | 6.0 |
| F | 14.2 | 4.5 | 7.7 |

**Table S4** Distances between nucleotides and key residues. ADP-occupied subunits are highlighted in gray. Measurements in Å are between the γ thiophosphate of ATPγS or β phosphate of ADP and the Cε of arginine or Cγ of glutamine.
